# Sex Differences in Preoperative Risk Profiles and 1-Year Mortality Following Elective Cardiac Surgery: A Retrospective Single-Centre Cohort Study

**DOI:** 10.3390/jcm15010059

**Published:** 2025-12-21

**Authors:** Caitlin Bozic, Magnus Strypet, Floor J. Mansvelder, Evert K. Jansen, Jennifer S. Breel, Henning Hermanns, Susanne Eberl

**Affiliations:** 1 Department of Anaesthesiology, Amsterdam University Medical Centres, 1105 AZ Amsterdam, The Netherlands; 2 Department of Cardiac Surgery, Amsterdam University Medical Centres, 1105 AZ Amsterdam, The Netherlands

**Keywords:** sex-specific risk profile, cardiac surgery, one-year mortality

## Abstract

**Background:** Sex-related differences in outcomes following cardiac surgery are well documented, with females generally experiencing higher postoperative mortality rates than males. However, the underlying factors driving this disparity remain incompletely understood. This study aimed to compare the preoperative risk characteristics of female and male patients who died within one year after elective cardiac surgery with those who survived, in order to identify sex-specific risk profiles associated with postoperative mortality. **Methods:** In this retrospective single-centre cohort study, data were derived from a prospective quality assurance database at Amsterdam University Medical Centres (Amsterdam UMC), The Netherlands, covering January 2001 to December 2020. All adult patients (≥18 years) undergoing elective cardiac surgery were included. Descriptive and comparative analyses were performed to characterise sex-specific preoperative differences between survivors and non-survivors. **Results:** The study cohort comprised 10,614 patients, including 2804 females (26%; median age 72 years [IQR 65–77]) and 7810 males (74%; median age 67 years [IQR 59–73]). In both sexes, non-survivors more frequently had major comorbidities, including atrial fibrillation, history of reoperation, pulmonary hypertension, chronic obstructive pulmonary disease, cerebrovascular disease, and kidney dysfunction. Within one year post-surgery, 143 (5.1%) females and 299 (3.8%) males had died. Among females, non-survivors within one year of surgery more frequently had several preoperative risk factors compared with survivors, including moderately impaired left ventricular function (16% vs. 11%), pulmonary hypertension (12% vs. 3%), extracardiac arteriopathy (25% vs. 9%), and kidney dysfunction (46% vs. 21%) dependent on the type of surgery (combined valve + coronary artery bypass grafting (CABG) (29% vs. 15%) or aortic surgery (14% vs. 4%)). In male patients, however, different risk factors such as higher age (median 73 years [IQR 66–77] vs. 67 [59–73]), lower Body Surface Area (mean 1.96 m^2^ (SD ± 0.19) vs. 2.02 ± 0.18), hypercholesterolaemia (35% vs. 44%), severely impaired left ventricular function (14% vs. 6%), myocardial infarction (31% vs. 22%), and type of surgery (aortic surgery (9% vs. 3%), or combined valve + CABG (22% vs. 12%)) were preoperative predictors of mortality compared to non-survivors. **Conclusions:** Our study demonstrates that one-year mortality following elective cardiac surgery is driven by distinct preoperative risk profiles in females and males. Recognising that mortality in females is associated with systemic disease and males by direct cardiac damage is a critical step toward developing more equitable, precise, and effective perioperative management strategies.

## 1. Introduction

Existing research consistently demonstrates that females undergoing cardiac surgery face a higher risk of mortality than males, both in the short and long term [1,2,3]. However, existing research often provides descriptive accounts of this disparity without clearly defining the preoperative causal factors that drive it. Commonly used preoperative risk prediction models, such as the EuroSCORE II, estimate surgical risk and support clinical decision-making before surgery [4]. Yet the inclusion of female sex as an independent risk factor in the EuroSCORE is problematic, as it reflects historical treatment differences rather than inherent biological susceptibility. Historically, female patients were older, referred later for intervention, and presented with smaller body size or more complex valve pathology, which collectively inflated their observed mortality rates. As a result, these models may encode a systemic bias that overestimates risk in contemporary cardiac surgery in female patients, potentially reinforcing inequities in access to appropriate surgical interventions. In essence, sex is treated as a static causal determinant and as a binary risk factor, with females assigned a higher baseline risk. This approach may obscure clinically relevant differences and limit opportunities for targeted optimisation in both sexes.

While age, comorbidities, and previous cardiac surgery [2,4,5,6] are recognised predictors of mortality, it remains unclear how the profile of these factors differ between surviving and non-surviving patients, specifically if stratified by sex. In particular, the specific preoperative characteristics of female and male patients who do not survive the first year after surgery are largely unknown. Identifying these patterns is crucial for refining risk assessment and guiding more personalised perioperative strategies.

Therefore, we conducted this retrospective cohort study at a Dutch academic medical centre and aimed to define and compare the preoperative risk profiles of female and male patients who died within one year of elective cardiac surgery. Secondary analyses aimed to develop sex-specific association models using logistic regression to identify key predictors of mortality in each group.

## 2. Materials and Methods

### 2.1. Study Design

This retrospective, single-centre, observational study analysed prospectively collected quality assurance data from all adult (≥18 years) patients who underwent elective cardiac surgery (coronary artery bypass grafting (CABG), aortic, valve or combined CABG/valve surgery) in the Amsterdam University Medical Centres (Amsterdam UMC), the Netherlands, between January 2001 and December 2020.

### 2.2. Ethical Considerations

On 10 February 2022, the Institutional Review Board of Amsterdam UMC confirmed that this study did not fall under the Medical Research involving Human Subjects Act and waived the need for individual patient consent (W22_050#22.081). This study was registered on ClinicalTrials.gov on 10 March 2022 (NCT06554925). This analysis adhered to the principles outlined in the Declaration of Helsinki (Helsinki, 2024) [7]. The Strengthening the Reporting of Observational Studies in Epidemiology (STROBE) guidelines were used in writing of this report [8].

### 2.3. Study Outcomes

The primary outcome was to characterise and compare the preoperative risk profile of female and male patients who died within one year following elective cardiac surgery with those of survivors. Secondary outcomes included developing sex-specific association models using logistic regression.

To evaluate one-year mortality, preoperative variables were analysed as potential risk factors if they showed differences between survivors and non-survivors within each sex. Variables demonstrating such differences were first identified and subsequently checked for sex-specificity. Variables unique to either sex were then included in the respective sex-specific risk profile. This stepwise approach allowed for the identification of preoperative factors associated with one-year mortality that were unique to females and males. Secondary analyses were performed to construct sex-specific logistic regression models. All preoperative variables identified as unique to either sex were considered candidates for inclusion. Model performance was evaluated using ROC curves, and the area under the curve (AUC) was calculated to assess the discriminative ability of each sex-specific model.

### 2.4. Data Sources and Measurements

All data were prospectively collected as part of a mandatory institutional quality assurance database. Data were verified after discharge by the same cardiac surgeon (EKJ) for the entire study period to ensure accuracy and completeness. Vital status was obtained through annual linkage to the national civil registry, which is linked to social security numbers, enabling reliable assessment of all-cause mortality within one year of surgery.

### 2.5. Definitions and Variables

All-cause mortality was defined as death from any cause, assessed cumulatively over the entire study period. One-year mortality referred to death within one year of the index surgery. Mortality within one year-after surgery was chosen as the primary outcome to include both early and intermediate deaths plausibly related to the surgical procedure and preoperative risk profile and allowing for comparison with previous studies.

Elective cardiac surgery included admissions for isolated or combined procedures involving CABG, valve, or aortic surgery.

A critical preoperative state was defined as hemodynamic instability requiring support such as inotropes, mechanical ventilation, or intra-aortic balloon pump. Reoperation was defined as surgery in patients with a history of previous cardiac surgery. A familial medical history referred to a documented family history of cardiovascular disease.

Left ventricular function (LVF) was categorised as normal: preserved systolic function; moderate dysfunction: moderately reduced ejection fraction; and severe dysfunction: severely reduced ejection fraction.

Infarct referred to myocardial infarction (MI), classified as ongoing: acute MI at the time of surgery or past 90 days: MI occurring within three months before surgery.

Pulmonary hypertension indicated elevated pulmonary arterial pressures (>25 mmHg). Extracardiac arteriopathy indicated the presence of clinically significant atherosclerotic disease in arteries outside the heart.

Neurological dysfunction referred to motor, sensory, or cognitive impairment. Kidney dysfunction was defined as an estimated glomerular filtration rate (eGFR) below 55 mL/min/1.73 m^2^.

### 2.6. Sample Size

No formal sample size calculation was performed. The study population comprised all patients who underwent elective cardiac surgery at Amsterdam UMC between January 2001 and December 2020. The total number of eligible patients during this period defined the sample size.

### 2.7. Statistical Analysis

Prior to analysis, data were checked for accuracy, missing data, outliers, and normality. The normality of continuous data was assessed by visual inspection of histograms, Q-Q plots, and boxplots. Statistical analysis was performed using R Studio version 4.4.3 (2025-02-28 ucrt).

Patient characteristics were presented using descriptive statistics. Normally distributed continuous data were presented as mean and standard deviation (SD). Non-normally distributed continuous data were presented as the median and interquartile range (IQR). Categorical variables were presented as numbers and percentages.

Sex-specific logistic regression models were used to determine the association of several preoperative variables and all-cause mortality within one year. All sex-specific variables were first entered into univariable logistic regression analyses, and those remaining significant were subsequently included in multivariable logistic regression models. Analyses were conducted separately for females and males. A *p*-value ≤ 0.05 was considered statistically significant.

## 3. Results

### 3.1. Study Population

**Figure 1:**
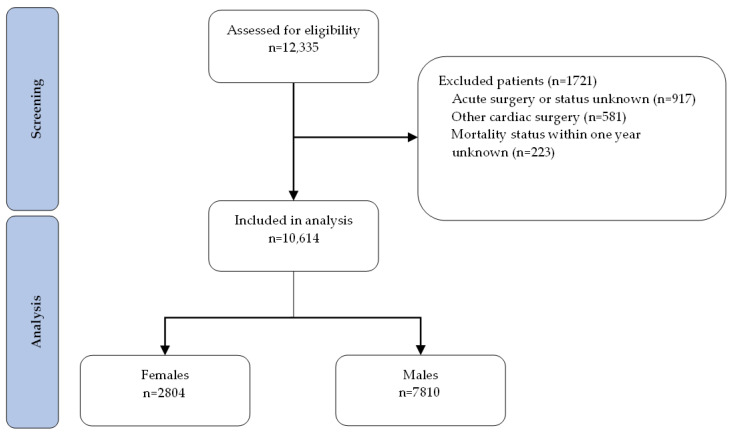
Flowchart patient inclusion.

Between January 2001 and December 2020, a total of 12,335 patients who underwent cardiac surgery at Amsterdam UMC were assessed for eligibility (Figure 1). Of these, 917 patients were excluded due to acute surgical indication or because the status was unknown, 581 because they had procedures outside the scope of interest (i.e., other than CABG, valve, valve + CABG or aortic surgery), and 223 due to unknown one-year postoperative mortality status. The final study cohort consisted of 10,614 patients, comprising 2804 females and 7810 males.

### 3.2. Patient Characteristics

#### 3.2.1. Females

Preoperative characteristics of female patients are presented in Table 1. Among the 2804 females included, 5.1% (*n* = 143) died within one year of surgery. The median age was 72 years [IQR 65–77], and the mean Body Surface Area (BSA) was 1.80 m^2^ (SD ± 0.17).

Compared with female survivors, female non-survivors more frequently had a history of reoperation (9% vs. 3%), atrial fibrillation (21% vs. 13%), moderate LVF (16% vs. 11%), pulmonary hypertension (12% vs. 3%), extracardiac arteriopathy (25% vs. 9%), chronic obstructive pulmonary disease (COPD) (18% vs. 10%), cerebrovascular accident/transient ischemic attack (CVA/TIA) (18% vs. 9%), and kidney dysfunction (46% vs. 21%). Non-survivors also had lower haemoglobin (median 7.8 mmol/L [IQR 7.1–8.4] vs. 8.1 mmol/L [7.6–8.6]). Surgical procedure type also differed; AVR was more common among non-survivors (50% vs. 38%).

#### 3.2.2. Males

Preoperative characteristics of male cardiac surgery patients are presented in Table 2. Of the 7810 males included, 3.8% (n = 299) died within one year of surgery. The median age was 67 years [IQR 59–73], and the mean BSA was 2.01 m^2^ (SD ± 0.18).

Compared with male survivors, male non-survivors were older (median age 73 years [IQR 66–77] vs. 67 years [59–73]) and had a smaller mean BSA (mean 1.96 m^2^ ± 0.19 vs. 2.02 ± 0.18 m^2^). They more frequently had a history of reoperation (10% vs. 4%), atrial fibrillation (23% vs. 11%), severe LVF (14% vs. 6%), myocardial infarction (31% vs. 22%), pulmonary hypertension (7% vs. 2%), COPD (17% vs. 8%), CVA/TIA (17% vs. 8%), and kidney dysfunction (35% vs. 9%).

Male non-survivors had lower median haemoglobin levels (median 8.2 mmol/L [IQR 7.3–8.9] vs. 8.9 mmol/L [8.3–9.4]) and were less likely to have a familial history of cardiovascular disease (23% vs. 30%). Procedural differences were also observed; AVR was more frequent among non-survivors (30% vs. 24%), whereas LIMA use was less common (61% vs. 73%).

### 3.3. Sex-Specific Preoperative Risk Profile

In total, 143 of 2804 females (5.1%) and 299 of 7810 males (3.8%) died within one year of elective cardiac surgery. Several risk factors were shared by both female and male non-survivors, including reoperation, smoking, COPD, CVA/TIA, and lower haemoglobin levels. These common factors were excluded from the sex-specific risk profiles. Given that the type of surgery is known preoperatively, we incorporated the distinct outcome profiles for each surgical type into the preoperative risk assessment.

#### 3.3.1. Females

**Figure 2:**
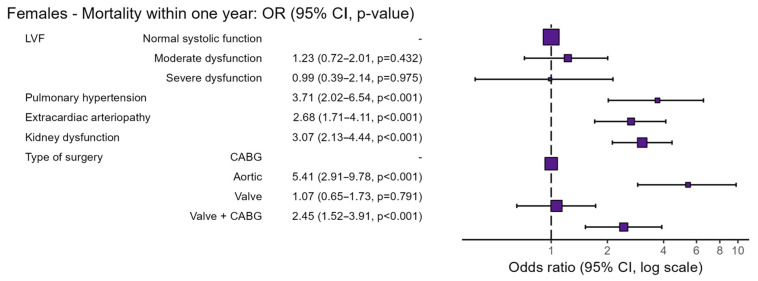
Preoperative risk factors of female patients undergoing cardiac surgery. Abbreviations: OR = odds ratio, CI = confidence interval, LVF = Left Ventricular Function, CABG = Coronary Artery Bypass Grafting.

Preoperative risk factors specific for female patients undergoing cardiac surgery are shown in Table 3 and Figure 2. In non-surviving females, moderate LVF was more prevalent (16% vs. 11%; OR 1.23, 95% CI 0.72–2.01, *p* = 0.432). Pulmonary hypertension was also more prevalent (12% vs. 3%; OR 3.71; 95% CI 2.02–6.54, *p* < 0.001), as were extracardiac arteriopathy (25% vs. 9%; OR 2.68; 95% CI 1.71–4.11, *p* < 0.001) and kidney dysfunction (46% vs. 21%; OR 3.07; 95% CI 2.13–4.44, *p* < 0.001). The type of surgery was another key determinant of mortality; non-surviving females more frequently underwent combined valve and CABG surgery (29% vs. 15%; OR 2.45; 95% CI 1.52–3.91, *p* < 0.001) and aortic surgery (14% vs. 4%; OR 5.41; 95% CI 2.91–9.78, *p* < 0.001).

**Figure 3:**
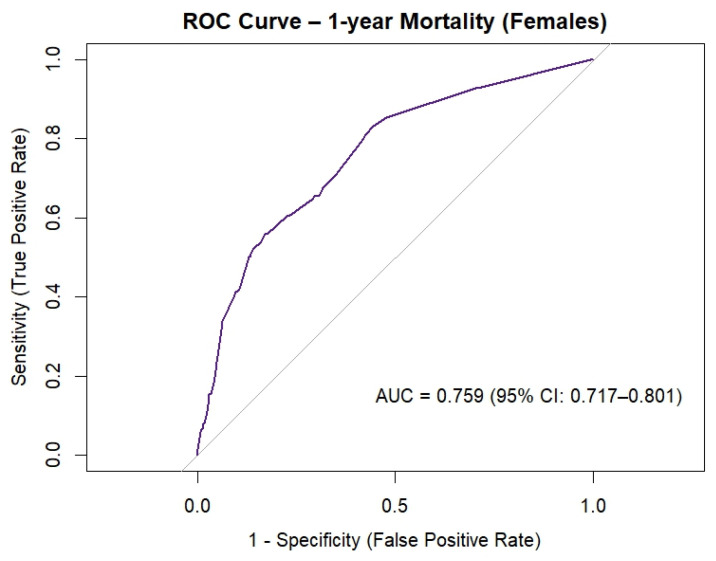
ROC curve of preoperative risk profile of female patients undergoing cardiac surgery. Abbreviations: AUC = area under the curve, CI = confidence interval.

Figure 3 shows the goodness of fit of the preoperative risk profile of female patients undergoing cardiac surgery, measured with the ROC curve. The AUC for the female risk profile was 0.759 (95% CI 0.717–0.801) and was considered acceptable.

#### 3.3.2. Males

**Figure 4:**
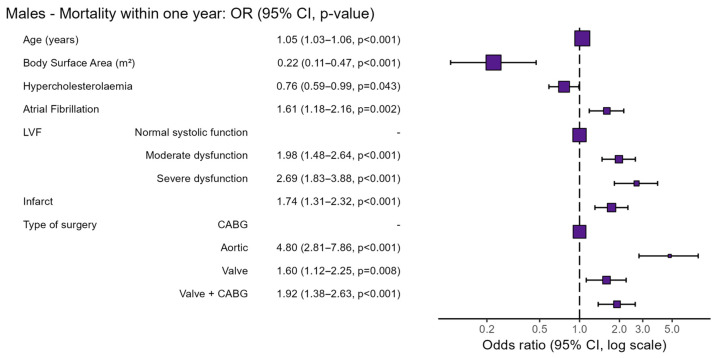
Preoperative risk factors of male patients undergoing cardiac surgery. Abbreviations: OR = odds ratio, CI = confidence interval, LVF = Left Ventricular Function, CABG = Coronary Artery Bypass Grafting.

Preoperative risk factors specific for male patients undergoing cardiac surgery are shown in Table 4 and Figure 4. Non-surviving males were older (median [IQR] 73 [66–77] vs. 67 [59–73]; OR 1.05; 95% CI 1.03–1.06, *p* < 0.001) and had lower BSA (1.96 m^2^ ± 0.19 vs. 2.02 m^2^ ± 0.18; OR 0.22; 95% CI 0.11–0.47, *p* < 0.001). Moreover, severe LVF was more prevalent among non-surviving males (14% vs. 6%; OR 2.69; 95% CI 1.83–3.88, *p* < 0.001), as was a history of myocardial infarction (31% vs. 22%; OR 1.74; 95% CI 1.31–2.32, *p* < 0.001). Conversely, hypercholesterolaemia was less prevalent in non-surviving males (35% vs. 44%; OR 0.76; 95% CI 0.59–0.99, *p* = 0.043).

Also, the type of surgery played a significant role as a risk factor; non-surviving males more frequently underwent a combination of valve and CABG surgery (22% vs. 12%; OR 1.92; 95% CI 1.38–2.63, *p* < 0.001) and aortic surgery (9% vs. 3%; OR 4.80; 95% CI 2.81–7.86, *p* < 0.001).

**Figure 5:**
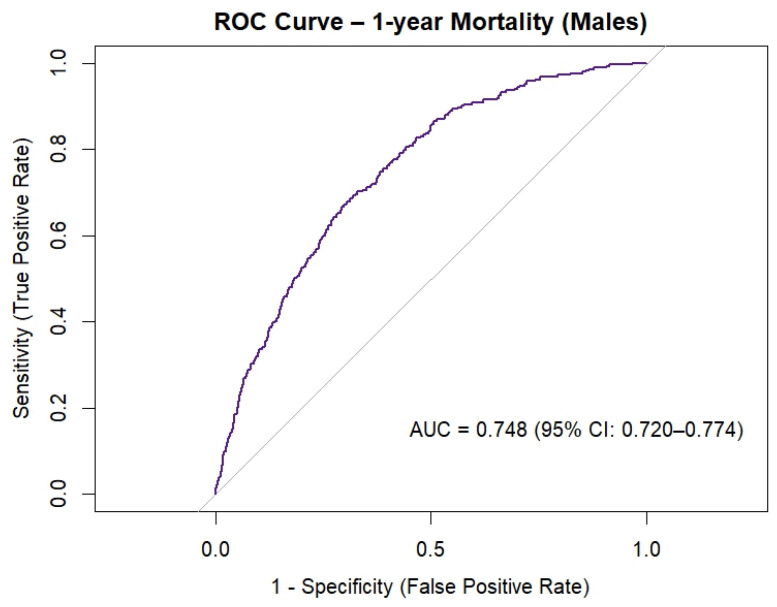
ROC curve of preoperative risk profile of male patients undergoing cardiac surgery. Abbreviations: AUC = area under the curve, CI = confidence interval.

Figure 5 shows the goodness of fit of the preoperative risk profile of male patients undergoing cardiac surgery, measured with the ROC curve. The AUC for the male preoperative risk profile was 0.748 (95% CI 0.720–0.774) and was considered acceptable.

### 3.4. Mortality over Time

**Figure 6:**
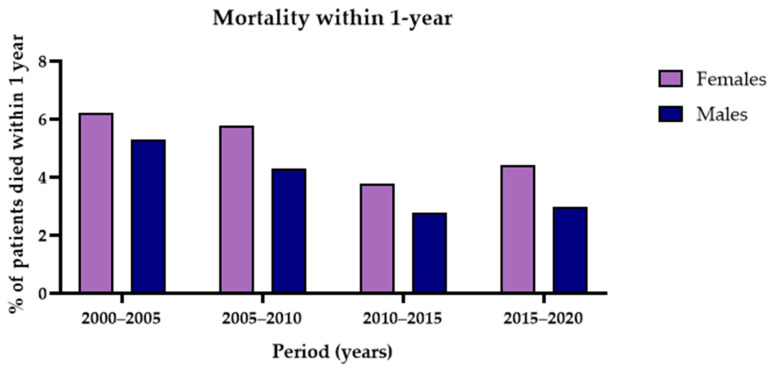
Mortality within one-year over time, for females and males during study period January 2001–December 2020.

This study was conducted between January 2001 and December 2020. Figure 6 illustrates the proportion of patients who died within one year of surgery, stratified by sex. Over the 20-year study period, one-year postoperative mortality decreased in both females and males.

## 4. Discussion

In this large, single-centre retrospective cohort study, we aimed to move beyond treating sex as a monolithic risk factor and instead characterise the distinct preoperative profiles associated with one-year mortality in females and males undergoing elective cardiac surgery. Our primary finding was that the risk factors for mortality differ significantly between the sexes. For female patients, one-year mortality was independently associated with a profile of systemic vascular and end-organ dysfunction, specifically pulmonary hypertension, extracardiac arteriopathy, and kidney dysfunction, as well as undergoing more complex procedures (combined valve/CABG or aortic surgery). In contrast, mortality in male patients was predominantly associated with older age, lower BSA, and markers of more advanced cardiac-specific damage, such as severe left ventricular dysfunction and a history of myocardial infarction. Both sex-specific models demonstrated acceptable discriminative ability, with AUCs of 0.759 for females and 0.748 for males, reinforcing the validity of a sex-differentiated approach to risk stratification.

Our findings in female patients contextualise and extend the existing literature, consistently reporting higher mortality for females after cardiac surgery [1,2,3]. While previous studies typically adjusted for sex as a binary covariate, our analysis reveals that these disparities stem from distinct underlying risk phenotypes. The female mortality profile, marked by moderate left ventricular function, pulmonary hypertension, extracardiac arteriopathy and kidney dysfunction, suggests advanced, systemic vasculopathy and multi-organ compromise. This aligns closely with modern evidence that females are more likely to present with coronary microvascular disease (CMD), particularly those with chronic comorbidities and heart failure with preserved ejection fraction (HFpEF), where CMD is highly prevalent and leads to increased mortality and hospitalisation risk [9]. Recent large studies show up to 75% of HFpEF patients, most of whom are female, exhibit CMD, often accompanied by pulmonary hypertension and systemic endothelial impairment, echoing the constellation we observed [10,11]. Additionally, CMD in these patients is linked with diastolic dysfunction and adverse outcomes, reinforcing the interdependence between microvascular disease, end-organ compromise, and increased surgical risk [10,11,12].

The male mortality profile, driven by older age, atrial fibrillation, severe LV dysfunction, and prior myocardial infarction, reflects a more “classic” ischemic cardiomyopathy pathway, which is well-documented in the cardiac surgery literature [2,5,6]. The association of lower BSA with higher mortality in males is also a key finding, likely serving as a proxy for frailty or sarcopenia, which are increasingly recognised as powerful independent predictors of poor postoperative outcomes [13]. Interestingly, our model identified hypercholesterolemia as a protective factor in males. This seemingly paradoxical finding has been observed in other large cohort studies and is often attributed to confounding by indication, wherein patients diagnosed with hypercholesterolaemia receive aggressive, long-term statin therapy, whose pleiotropic anti-inflammatory and plaque-stabilising effects confer a survival benefit that outweighs the risk of the diagnosis itself [14].

While prior studies have investigated preoperative risk profiles for patients undergoing cardiac surgery, most models pool males and females and apply a single coefficient for sex rather than deriving separate, sex-specific profiles. For instance, EuroSCORE II incorporates sex as a binary variable and assigns higher baseline risk to female patients [4]. Similarly, the Society of Thoracic Surgeons (STS)’ risk calculator treats sex dichotomously, attributing higher operative and postoperative mortality risk to females [15]. While these models acknowledge sex differences, they do not account for sex-specific variations in the prevalence, interactions, or effect sizes of individual predictors. The present findings demonstrate that several preoperative factors exert differential associations with one-year mortality in females versus males, underscoring a key limitation of existing models and supporting the development of sex-specific preoperative risk assessment that moves beyond a uniform “female penalty”.

### 4.1. Limitations

The study has important limitations. Its retrospective, single-centre design makes the findings descriptive and susceptible to selection bias and unmeasured confounding, reducing generalizability to other populations and health systems. Only preoperative variables were analysed, so intraoperative and postoperative factors that materially affect outcomes were not captured. Although surgical teams were largely consistent, advances in surgical technique, perioperative care, and technology over the 20-year period likely influenced mortality and complication rates. Exclusion of acute (urgent/emergent) cases further narrows applicability. Key confounders—socioeconomic status, medication adherence, and detailed frailty metrics—were unavailable, and the database lacked granular echocardiographic measures (notably left ventricular diastolic function and right ventricular function), which may partly account for the observed sex differences, especially in females. Given the known relevance in postoperative outcome in case of diastolic dysfunction and heart failure with preserved ejection fraction (HFpEF) particularly in females, this lack of detailed functional data may have led to residual confounding [16]. Finally, emphasising sex-specific rather than shared risk factors highlights disparities but may underrepresent an individual patient’s total risk burden.

### 4.2. Strengths

This study demonstrates several key strengths that support its validity and relevance. By including all patients undergoing cardiac surgery at a single tertiary care centre over a 20-year period, it provides a large, representative sample while reducing the risk of selection bias. Data collection was standardised, and thorough post-discharge review by the same surgeon (EKJ) throughout the study ensured consistency and accuracy. Mortality outcomes were confirmed using the Dutch database for registers of persons (Basisregistratie Personen) as of 1 November 2023, guaranteeing complete follow-up and minimising the risk of missing data. These careful procedures contributed to a robust dataset with very few errors or omissions.

### 4.3. Future Perspectives

Looking forward, these findings advocate for a shift toward more personalised, sex-aware perioperative risk assessment. Clinicians should be particularly vigilant for markers of systemic end-organ damage (pulmonary hypertension, extracardiac arteriopathy, kidney dysfunction) when evaluating female patients, as these may signal a higher-risk phenotype that is not fully captured by traditional cardiac-focused scores. Future research should focus on validating these sex-specific risk models in multicentre, external cohorts. Prospective studies are also warranted to investigate the underlying mechanisms of these distinct risk profiles, potentially incorporating biomarkers of inflammation, fibrosis, and detailed assessments of frailty and microvascular function. It is equally important to integrate sex-specific considerations into clinical decision-making and guideline development. Tailored preoperative assessment tools, targeted risk mitigation strategies, and heightened awareness of sex-related vulnerabilities may improve outcomes for both female and male patients. Moreover, incorporating sex-specific reporting standards, similar to the Sex and Gender Equity in Research (SAGER) guidelines in research, could enhance transparency and reproducibility, fostering a more nuanced understanding of how sex influences surgical risk and recovery in cardiac surgery.

## 5. Conclusions

In conclusion, our study demonstrates that one-year mortality following elective cardiac surgery is driven by distinct preoperative risk profiles in females and males. Recognising that mortality in females is associated with systemic disease and in males with direct cardiac damage is a critical step toward developing more equitable, precise, and effective perioperative management strategies.

## Figures and Tables

**Table 1:** Patient characteristics of all female patients.

	Total (*n* = 2804)	Alive (*n* = 2661)	Dead (*n* = 143)
Age (years)	72 [65–77]	71 [65–76]	74 [69–80]
Height (cm)	164 ± 7	164 ± 7	163 ± 7
Weight (kg)	72.0 [64.0–82.0]	72.0 [64.0–82.0]	69.5 [60.0–78.3]
Body Mass Index (kg/m^2^)	26.7 [23.9–30.1]	26.7 [23.9–30.1]	26.2 [23.3–29.8]
Body Surface Area (m^2^)	1.80 ± 0.17	1.80 ± 0.17	1.76 ± 0.19
**General**			
Critical preoperative state	37 (1%)	33 (1%)	4 (2%)
NTG IV	155 (5%)	142 (5%)	13 (9%)
IABP	31 (1%)	31 (1%)	0 (0%)
Euroscore	6.00 [4.00–8.00]	6.00 [4.00–8.00]	8.00 [6.50–10.0]
Euroscore II	2.24 [1.44–3.97]	2.18 [1.41–3.74]	5.18 [2.69–8.80]
Log euroscore	5.13 [3.13–8.47]	5.09 [3.05–8.29]	10.1 [6.26–16.2]
Reoperation	107 (3%)	94 (3%)	13 (9%)
Previous aortic surgery	93 (3%)	84 (3%)	9 (6%)
Previous CABG surgery	53 (1%)	44 (1%)	9 (6%)
Previous valve surgery	46 (1%)	43 (1%)	3 (2%)
**Medical history**			
Familial medical history	886 (31%)	846 (31%)	40 (28%)
Diabetes Mellitus	579 (20%)	546 (20%)	33 (23%)
Hypertension	1643 (58%)	1559 (58%)	84 (58%)
Hypercholesterolaemia	1133 (40%)	1074 (40%)	59 (41%)
Smoking	359 (12%)	335 (12%)	24 (16%)
Thyroid disease	188 (6%)	173 (6%)	15 (10%)
**Cardiovascular medical history**			
Atrial Fibrillation	398 (14%)	368 (13%)	30 (21%)
LVF			
Normal systolic function	2302 (85%)	2193 (85%)	109 (79%)
Moderate dysfunction	308 (11%)	286 (11%)	22 (16%)
Severe dysfunction	96 (4%)	89 (4%)	7 (5%)
PTCA	368 (13%)	347 (13%)	21 (14%)
Infarct	389 (13%)	367 (13%)	22 (15%)
Infarct ongoing	8 (0%)	8 (0%)	0 (0%)
Infarct past 90 days	317 (11%)	299 (11%)	18 (12%)
Pulmonary hypertension	122 (4%)	104 (3%)	18 (12%)
Extracardiac arteriopathy	276 (9%)	240 (9%)	36 (25%)
**Pulmonary medical history**			
COPD	304 (10%)	277 (10%)	27 (18%)
**Neurological medical history**			
CVA/TIA	276 (9%)	250 (9%)	26 (18%)
Neurological dysfunction	62 (2%)	54 (2%)	8 (5%)
**Kidney medical history**			
Kidney dysfunction	634 (22%)	568 (21%)	66 (46%)
Dialysis	12 (1%)	10 (1%)	2 (1%)
**Preoperative laboratory**			
CKD-EPI eGFR (mL/min/1.72 m^2^)	68.0 [55.0–83.5]	68.0 [56.0–84.0]	57.5 [41.0–71.0]
Haemoglobin (mmol/L)	8.1 [7.6–8.6]	8.1 [7.6–8.6]	7.8 [7.1–8.4]
CRP (mg/L)	3.00 [2.50–7.00]	3.00 [2.50–7.00]	5.00 [2.50–11.0]
ESR (mm)	17.0 [9.00–28.0]	17.0 [9.00–28.0]	25.0 [14.0–38.5]
Leucocyte count (×10^9^/L)	7.60 [6.30–9.00]	7.50 [6.30–9.00]	8.00 [6.40–9.50]
Platelet count (×10^9^/L)	259 [216–309]	260 [217–309]	249 [197–308]
aPTT (seconds)	35.0 [32.0–40.0]	35.0 [32.0–40.0]	37.0 [33.0–44.0]
INR	1.02 [0.970–1.09]	1.02 [0.970–1.08]	1.05 [1.01–1.23]
**Surgery**			
Weight intervention			
isolated CABG	1289 (46%)	1245 (47%)	44 (31%)
single non-CABG	874 (31%)	840 (31%)	34 (24%)
2 procedures	530 (19%)	482 (18%)	48 (33%)
3 procedures	111 (4%)	94 (4%)	17 (12%)
Type of surgery			
CABG	1281 (46%)	1237 (46%)	44 (31%)
Aortic	128 (5%)	108 (4%)	20 (14%)
Valve	965 (34%)	927 (35%)	38 (26%)
Valve + CABG	430 (15%)	389 (15%)	41 (29%)
AVR	1086 (38%)	1014 (38%)	72 (50%)
MVR	463 (16%)	436 (16%)	27 (18%)
TVR	72 (2%)	66 (2%)	6 (4%)
LIMA	1494 (53%)	1424 (53%)	70 (49%)
RIMA	213 (7%)	200 (7%)	13 (9%)
Off-pump surgery	296 (10%)	283 (10%)	13 (9%)

Data shown as mean ± SD, median [IQR], or numbers (%). Abbreviations: NTG IV = Intravenous Nitroglycerin, IABP = Intra-Aortic Balloon Pump, CABG = Coronary Artery Bypass Grafting, LVF = Left Ventricular Function, PTCA = Percutaneous Transluminal Coronary Angioplasty, LMCA = Left Main Coronary Artery, COPD = Chronic Obstructive Pulmonary Disease, CVA/TIA = Cerebrovascular Accident/Transient Ischemic Attack, CKD-EPI eGFR = Chronic Kidney Disease Epidemiology Collaboration estimated Glomerular Filtration Rate, CRP = C-Reactive Protein, ESR = Erythrocyte Sedimentation Rate, aPTT = Activated Partial Thromboplastin Time, INR = International Normalised Ratio, AVR = Aortic Valve Replacement, MVR = Mitral Valve Replacement, TVR = Tricuspid Valve Replacement, TAVI = Transcatheter Aortic Valve Implantation, LIMA = Left Internal Mammary Artery, RIMA = Right Internal Mammary Artery. Missing values: Height: 57 (2%); Weight: 47 (2%); Body Mass Index: 57 (2%); Body Surface Area: 57 (2%); Atrial Fibrillation: 4 (0%); LVF: 98 (3%); Kidney dysfunction: 12 (0%); CKD-EPI eGFR: 65 (2%); Haemoglobin: 109 (4%); CRP: 153 (5%); ESR: 327 (12%); Leucocyte count: 119 (4%); Platelet count: 127 (5%); aPTT: 136 (5%); INR: 136 (5%).

**Table 2:** Patient characteristics of all male patients.

	Total (*N* = 7810)	Alive (*n* = 7511)	Dead (*n* = 299)
Age (years)	67 [59–73]	67 [59–73]	73 [66–77]
Height (cm)	177 ± 8	177 ± 8	175 ± 8
Weight (kg)	84.0 [75.0–93.0]	84.0 [75.0–93.0]	79.0 [72.0–90.0]
Body Mass Index (kg/m^2^)	26.7 [24.6–29.3]	26.8 [24.6–29.4]	25.9 [23.7–29.1]
Body Surface Area (m^2^)	2.01 ± 0.18	2.02 ± 0.18	1.96 ± 0.19
**General**			
Critical preoperative state	92 (1%)	74 (1%)	18 (6%)
NTG IV	373 (4%)	343 (4%)	30 (10%)
IABP	59 (0%)	54 (0%)	5 (1%)
Euroscore	4.00 [3.00–6.00]	4.00 [3.00–6.00]	7.00 [5.00–9.00]
Euroscore II	1.47 [0.943–2.56]	1.44 [0.930–2.48]	3.19 [1.86–7.23]
Log euroscore	2.71 [1.51–5.24]	2.66 [1.51–5.11]	6.81 [3.77–13.0]
Reoperation	347 (4%)	315 (4%)	32 (10%)
Previous aortic surgery	335 (4%)	302 (4%)	33 (11%)
Previous CABG surgery	218 (2%)	198 (2%)	20 (6%)
Previous valve surgery	114 (1%)	102 (1%)	12 (4%)
**Medical history**			
Familial medical history	2390 (30%)	2318 (30%)	72 (24%)
Diabetes Mellitus	1540 (19%)	1467 (19%)	73 (24%)
Hypertension	3983 (51%)	3829 (51%)	154 (51%)
Hypercholesterolaemia	3473 (44%)	3366 (44%)	107 (35%)
Smoking	1422 (18%)	1359 (18%)	63 (21%)
Thyroid disease	138 (1%)	130 (1%)	8 (2%)
**Cardiovascular medical history**			
Atrial Fibrillation	919 (11%)	849 (11%)	70 (23%)
LVF			
Normal systolic function	5834 (77%)	5661 (78%)	173 (59%)
Moderate dysfunction	1251 (16%)	1173 (16%)	78 (27%)
Severe dysfunction	474 (6%)	434 (6%)	40 (14%)
PTCA	1253 (16%)	1205 (16%)	48 (16%)
Infarct	1754 (22%)	1660 (22%)	94 (31%)
Infarct ongoing	17 (0%)	12 (0%)	5 (1%)
Infarct past 90 days	1321 (16%)	1265 (16%)	56 (18%)
Pulmonary hypertension	211 (2%)	188 (2%)	23 (7%)
Extracardiac arteriopathy	872 (11%)	804 (10%)	68 (22%)
**Pulmonary medical history**			
COPD	694 (8%)	643 (8%)	51 (17%)
**Neurological medical history**			
CVA/TIA	682 (8%)	630 (8%)	52 (17%)
Neurological dysfunction	206 (2%)	186 (2%)	20 (6%)
**Kidney medical history**			
Kidney dysfunction	818 (10%)	712 (9%)	106 (35%)
Dialysis	52 (1%)	43 (1%)	9 (3%)
**Preoperative laboratory**			
CKD-EPI eGFR (mL/min/1.72 m^2^)	77.0 [64.0–91.0]	77.0 [64.0–91.0]	60.0 [43.0–76.5]
Haemoglobin (mmol/L)	8.9 [8.2–9.4]	8.9 [8.3–9.4]	8.2 [7.3–8.9]
CRP (mg/L)	2.50 [2.50–7.00]	2.50 [2.50–6.00]	6.00 [2.50–15.0]
ESR (mm)	9.00 [4.00–19.0]	9.00 [4.00–18.0]	20.0 [10.0–36.0]
Leucocyte count (×10^9^/L)	7.60 [6.30–8.90]	7.60 [6.30–8.90]	8.10 [6.60–9.80]
Platelet count (×10^9^/L)	231 [195–275]	231 [195–275]	228 [183–281]
aPTT (seconds)	36.0 [33.0–40.0]	36.0 [33.0–40.0]	38.0 [34.0–46.0]
INR	1.03 [0.990–1.10]	1.03 [0.990–1.09]	1.07 [1.01–1.26]
**Surgery**			
Weight intervention			
isolated CABG	5263 (67%)	5105 (68%)	158 (53%)
single non-CABG	1287 (16%)	1235 (16%)	52 (17%)
2 procedures	1033 (13%)	968 (13%)	65 (22%)
3 procedures	227 (3%)	203 (3%)	24 (8%)
Type of surgery			
CABG	5249 (67%)	5096 (68%)	153 (51%)
Aortic	267 (3%)	241 (3%)	26 (9%)
Valve	1347 (17%)	1292 (17%)	55 (18%)
Valve + CABG	947 (12%)	882 (12%)	65 (22%)
AVR	1879 (24%)	1784 (23%)	95 (31%)
MVR	694 (8%)	644 (8%)	50 (16%)
TVR	57 (0%)	50 (0%)	7 (2%)
LIMA	5687 (72%)	5502 (73%)	185 (61%)
RIMA	960 (12%)	941 (12%)	19 (6%)
Off-pump surgery	1185 (15%)	1150 (15%)	35 (11%)

Data shown as mean ± SD, median [IQR], or numbers with %. Abbreviations: NTG IV = Intravenous Nitroglycerin, IABP = Intra-Aortic Balloon Pump, CABG = Coronary Artery Bypass Grafting, LVF = Left Ventricular Function, PTCA = Percutaneous Transluminal Coronary Angioplasty, LMCA = Left Main Coronary Artery, COPD = Chronic Obstructive Pulmonary Disease, CVA/TIA = Cerebrovascular Accident/Transient Ischemic Attack, CKD-EPI eGFR = Chronic Kidney Disease Epidemiology Collaboration estimated Glomerular Filtration Rate, CRP = C-Reactive Protein, ESR = Erythrocyte Sedimentation Rate, aPTT = Activated Partial Thromboplastin Time, INR = International Normalised Ratio, AVR = Aortic Valve Replacement, MVR = Mitral Valve Replacement, TVR = Tricuspid Valve Replacement, TAVI = Transcatheter Aortic Valve Implantation, LIMA = Left Internal Mammary Artery, RIMA = Right Internal Mammary Artery. Missing: Height: 165 (2%); Weight: 140 (2%); Body Mass Index: 165 (2%); Body Surface Area: 164 (2%); Euroscore: 738 (9%); Euroscore II: 1 (0%); Atrial Fibrillation: 17 (0%); LVF: 251 (3%); Kidney dysfunction: 52 (1%); CKD-EPI eGFR 180 (2%); Haemoglobin: 262 (3%); CRP: 409 (5%); ESR: 822 (11%); Leucocyte count: 300 (4%); Platelet count: 315 (4%); aPTT: 330 (4%); INR: 355 (5%).

**Table 3:** Logistic regression results of elective females and mortality within one year.

Variables		Alive	Dead	Univariable—OR (95% CI)	Multivariable—OR (95% CI)
LVF	Normal systolic function	2193 (85%)	109 (79%)	-	-
	Moderate dysfunction	286 (11%)	22 (16%)	1.55 (0.94–2.44, *p* = 0.071)	1.23 (0.72–2.01, *p* = 0.432)
	Severe dysfunction	89 (4%)	7 (5%)	1.58 (0.65–3.27, *p* = 0.257)	0.99 (0.39–2.14, *p* = 0.975)
Pulmonary hypertension		104 (3%)	18 (12%)	3.54 (2.02–5.89, *p* < 0.001)	3.71 (2.02–6.54, *p* < 0.001)
Extracardiac arteriopathy		240 (9%)	36 (25%)	3.39 (2.25–5.02, *p* < 0.001)	2.68 (1.71–4.11, *p* < 0.001)
Kidney dysfunction		568 (21%)	66 (46%)	3.23 (2.28–4.55, *p* < 0.001)	3.07 (2.13–4.44, *p* < 0.001)
Type of surgery	CABG	1237 (46%)	44 (31%)	-	-
	Aortic	108 (4%)	20 (14%)	5.21 (2.91–9.04, *p* < 0.001)	5.41 (2.91–9.78, *p* < 0.001)
	Valve	927 (35%)	38 (26%)	1.15 (0.74–1.79, *p* = 0.530)	1.07 (0.65–1.73, *p* = 0.791)
	Valve + CABG	389 (15%)	41 (29%)	2.96 (1.90–4.61, *p* < 0.001)	2.45 (1.52–3.91, *p* < 0.001)

Data shown as mean ± SD, median [IQR], or numbers with %. Abbreviations: OR = odds ratio, CI = confidence interval, LVF = Left Ventricular Function, CABG = Coronary Artery Bypass Grafting.

**Table 4:** Logistic regression results of elective males and mortality within one year.

Variables		Alive	Dead	Univariable—OR (95% CI)	Multivariable—OR (95% CI)
Age (years)	Median [IQR]	67 [59–73]	73 [66–77]	1.06 (1.05–1.07, *p* < 0.001)	1.05 (1.03–1.06, *p* < 0.001)
Body Surface Area (m^2^)	Mean ± SD	2.02 ± 0.18	1.96 ± 0.19	0.16 (0.08–0.32, *p* < 0.001)	0.22 (0.11–0.47, *p* < 0.001)
Hypercholesterolaemia		3366 (44%)	107 (35%)	0.69 (0.54–0.87, *p* = 0.002)	0.76 (0.59–0.99, *p* = 0.043)
Atrial Fibrillation		849 (11%)	70 (23%)	2.39 (1.80–3.14, *p* < 0.001)	1.61 (1.18–2.16, *p* = 0.002)
LVF	Normal systolic function	5661 (78%)	173 (59%)	-	-
	Moderate dysfunction	1173 (16%)	78 (27%)	2.18 (1.65–2.85, *p* < 0.001)	1.98 (1.48–2.64, *p* < 0.001)
	Severe dysfunction	434 (6%)	40 (14%)	3.02 (2.08–4.27, *p* < 0.001)	2.69 (1.83–3.88, *p* < 0.001)
Infarct		1660 (22%)	94 (31%)	1.62 (1.25–2.07, *p* < 0.001)	1.74 (1.31–2.32, *p* < 0.001)
Type of surgery	CABG	5096 (68%)	153 (51%)	-	-
	Aortic	241 (3%)	26 (9%)	3.59 (2.28–5.46, *p* < 0.001)	4.80 (2.81–7.86, *p* < 0.001)
	Valve	1292 (17%)	55 (18%)	1.42 (1.03–1.93, *p* = 0.029)	1.60 (1.12–2.25, *p* = 0.008)
	Valve + CABG	882 (12%)	65 (22%)	2.45 (1.81–3.29, *p* < 0.001)	1.92 (1.38–2.63, *p* < 0.001)

Data shown as mean ± SD, median [IQR], or numbers with %. Abbreviations: OR = odds ratio, CI = confidence interval, LVF = Left Ventricular Function, CABG = Coronary Artery Bypass Grafting.

## Data Availability

Data used in the writing of this manuscript is directly identifiable and part of an ongoing study and will therefore not be made available for data sharing.

## Abbreviations

The following abbreviations are used in this manuscript:

CABG	Coronary Artery Bypass Grafting
STROBE	Strengthening the Reporting of Observational Studies in Epidemiology
AUC	Area Under the Curve
IABP	Intra-Aortic Balloon Pump
LVF	Left Ventricular Function
MI	Myocardial Infarction
eGFR	Estimated Glomerular Filtration Rate
SD	Standard Deviation
IQR	Interquartile Range
BSA	Body Surface Area
COPD	Chronic Obstructive Pulmonary Disease
CVA/TIA	Cerebrovascular Accident/Transient Ischemic Attack
PTCA	Percutaneous Transluminal Coronary Angioplasty
NTG IV	Intravenous Nitroglycerin
LMCA	Left Main Coronary Artery
CRP	C-Reactive Protein
ESR	Erythrocyte Sedimentation Rate
aPTT	Activated Partial Thromboplastin Time
INR	International Normalised Ratio
AVR	Aortic Valve Replacement
MVR	Mitral Valve Replacement
TVR	Tricuspid Valve Replacement
TAVI	Transcatheter Aortic Valve Implantation
LIMA	Left Internal Mammary Artery
RIMA	Right Internal Mammary Artery
OR	Odds Ratio
CI	Confidence Interval
CMD	Coronary Microvascular Disease
HFpEF	Heart Failure with Preserved Ejection Fraction
